# Risk factors for the population’s mental health amidst the COVID-19 pandemic

**DOI:** 10.1590/1980-220X-REEUSP-2022-0324en

**Published:** 2023-06-23

**Authors:** Mariana Ferreira Vale, Elton Brás Camargo-Júnior, Fernando Augusto Cintra Magalhães, José Jailson de Almeida-Júnior, Edilaine Cristina da Silva Gherardi-Donato, Maria Neyrian de Fátima Fernandes

**Affiliations:** 1 Universidade Federal do Maranhão, Curso de Enfermagem, Imperatriz, MA, Brazil.; 2 Universidade de Rio Verde, Escola de Enfermagem, Rio Verde, GO, Brazil.; 3 Universidade Federal do Rio Grande do Norte, Departamento de Saúde Coletiva, Natal, RN, Brazil.; 4 Universidade de São Paulo, Escola de Enfermagem de Ribeirão Preto, Departamento de Enfermagem Psiquiátrica e Ciências Humanas, Ribeirão Preto, SP, Brazil.

**Keywords:** Coronavirus Infection, COVID-19, Stress, Psychological, Mindfulness, Nursing, Mental Health, Infecciones por Coronavirus, COVID-19, Estrés Psicológico, Atención Plena, Enfermería, Salud Mental, Infecções por Coronavírus, COVID-19, Estresse Psicológico, Atenção Plena, Enfermagem, Saúde Mental

## Abstract

**Objective:**

To identify risk factors for mental health in the population in times of COVID-19 through the analysis of levels of socio-cognitive mindfulness and perception of stress in individuals.

**Method:**

This is a cross-sectional observational study with a quantitative approach, carried out through online data collection using the Perceived Stress Scale and the Langer Mindfulness Scale in a sample of 955 individuals from different regions of Brazil.

**Results:**

Women, younger people and individuals with low socioeconomic conditions had higher levels of perceived stress; on the other hand, older men and individuals with high socioeconomic status had higher levels of mindfulness.

**Conclusion:**

Socio-cognitive mindfulness was not a protective factor for perceived stress in the context of the COVID-19 pandemic.

## RESUMO

**Objetivo:**

Identificar fatores de risco para a saúde mental na população em tempos de COVID-19 por meio da análise dos níveis atenção plena sociocognitiva e de percepção ao estresse nos indivíduos.

**Método:**

Trata-se de um estudo observacional do tipo transversal com abordagem quantitativa, realizado por meio de coleta de dados *online* utilizando a Escala de Estresse Percebido e a Escala de Atenção Plena de Langer em uma amostra de 955 indivíduos de diversas regiões do Brasil.

**Resultados:**

Mulheres, pessoas mais jovens e indivíduos com baixas condições socioeconômicas apresentaram maiores níveis de estresse percebido. Em contrapartida, os homens mais velhos e indivíduos com altas condições socioeconômicas apresentaram maiores níveis de atenção plena.

**Conclusão:**

A atenção plena sociocognitiva não se constituiu fator de proteção ao estresse percebido no contexto pandêmico da COVID-19.

## RESUMEN

**Objetivo:**

Identificar factores de riesgo para la salud mental en la población en tiempos de COVID-19 a través del análisis de los niveles de mindfulness sociocognitivo y percepción de estrés en los individuos.

**Método:**

Se trata de un estudio observacional transversal con enfoque cuantitativo, realizado a través de la recolección de datos en línea utilizando la Escala de Estrés Percibido y la Escala de Mindfulness de Langer en una muestra de 955 individuos de diferentes regiones de Brasil.

**Resultados:**

Las mujeres, los jóvenes y las personas con condiciones socioeconómicas bajas presentaron mayores niveles de estrés percibido. Por el contrario, los hombres mayores y las personas con un nivel socioeconómico alto tenían niveles más altos de atención plena.

**Conclusión:**

El mindfulness sociocognitivo no fue un factor protector para el estrés percibido en el contexto de la pandemia de COVID-19.

## INTRODUCTION

In December 2019 in Hubei province, China, the first cases of pneumonia of unknown cause, the new coronavirus (COVID-19), were reported, causing the authorities of that country to begin an investigation to characterize and control the disease^(1)^. Among the control measures adopted were social isolation of suspected cases, monitoring of contacts and collection of clinical and epidemiological data of patients^(1)^. In January 2020, the Chinese government released a notice specifying that psychological crisis intervention would also be part of the public health response to the outbreak^(2)^.

Despite the measures taken, the new disease quickly spread to 114 countries, leading the World Health Organization (WHO) to officially declare the global pandemic scenario on March 11, 2020^(3)^. In Brazil, the first case of COVID-19 was registered on February 26, in São Paulo. A month later, all states had confirmed cases, and the country officially registered 77 deaths from COVID-19^(4)^.

The COVID-19 pandemic decree placed the world in front of a new disease with a high contagion power and the uncertainties arising from the lack of a safe and effective treatment. Thus, COVID-19 caused profound transformations, spreading rapidly through the interconnections of a globalized world, reaching these interconnections and demanding a rapid reorganization of the various sectors of society to understand, adapt and combat the new virus. In this context, the restriction of mobility adopted in several countries, economic uncertainties, fear, daily deaths from the disease and the explosion of information to cover the local and global situation became part of everyday life^(5)^.

Taking into account this context of transformations, fears and uncertainties, the United Nations recognized that the crisis caused by the pandemic had the seeds for a major mental health crisis if no action was taken, therefore, the need to prioritize actions that promote support for individuals to deal with this moment^(6)^. The need to deal with uncertainty and fear of crisis are risk factors for the development of various mental disorders, from loss of productivity to suicide. In this context, fear of the pandemic causes anxiety and stress, directly affecting the way people perceive the events around them.

Crisis situations such as the COVID-19 pandemic have the potential to cause widespread damage that requires society to be prepared and resilient. Thus, with the aim of contributing to the instrumentalization of society to deal with these situations, we consider it important to know how individuals were affected by the pandemic and their ability to face the transformations resulting from the crisis. The present study aimed to identify risk factors for mental health in times of COVID-19 in the general population.

## METHODS

### Study Design

This is a cross-sectional observational study with a quantitative approach. The survey was conducted online based on the Strengthening the Reporting of Observational Studies in Epidemiology (STROBE) and Checklist for Reporting Results of Internet E-Surveys (CHERRIES) protocols.

### Population and Sample

It was intended to involve the entire population residing in Brazil. The sample was self-selected by the general population, composed according to the accessibility or convenience criterion, thus constituting a non-probabilistic sample of volunteers who spontaneously answered the questionnaires. In total, 955 people from different Brazilian states participated in the study.

### Selection Criteria

The accessibility selection criterion was used, and people were approached through social networks, emails, the university’s academic system and cell phone applications. Volunteers over 18 years old, with internet access and the ability to read and understand the questionnaire available in Portuguese were included. In order to minimize the bias of the online survey, the necessary care was taken when allocating the questionnaire to different groups, using different dissemination tools. Participants who failed to respond to at least 20% of items in each questionnaire and with a very short questionnaire response time (up to two minutes) were excluded.

### Data Collection

Data collection took place from September 2020 to January 2021. Disclosure was made on the social networks of research groups, students, pregnant women, athletes from all over the country. The link with access to the instruments was made available in the information systems and social networks of the *Universidade Federal do Maranhão* (UFMA). In all, that link received 1,927 clicks. Thus, the link consisted of self- administered instruments: a sociodemographic and health questionnaire prepared for this study, as well as the validated versions for the Brazilian context of the Perceived Stress Scale (PSS)^(7)^ and the Langer Mindfulness Scale (LMS)^(8)^. The questions did not have mandatory answers as recommended by the CHERRIES protocol.

The sociodemographic questionnaire consisted of questions related to gender, age, marital status, education, employment relationship, type of work (remote or face-to-face), skin color (white or non-white), religious belief, family income (in minimum wages) and children. Questions related to health data, whether the participant had or lived with someone with a chronic illness, whether participant or family member had COVID-19, whether someone close (family, friends, neighbors) died as a result of COVID-19, frequency of consumption of alcoholic beverages, psychotherapeutic follow-up (yes/no) and psychotropic treatment (yes/no and which one). The questions sought to know information about the moment of the pandemic. The exception was about the feeling of overload (work and daily activities), for which a comparison with the previous period of the pandemic was requested through the response on a numerical scale ranging from 1 to 4 (1 = totally agree to 4 = totally disagree) to the following statement: I feel more overwhelmed today than before social isolation.

The PSS, elaborated by Cohen et al.^(9)^, is a public domain instrument that determines the degree of perception of situations considered stressful^(9)^. The version used in this study consists of 14 items, translated from American English into Portuguese and validated in Brazil^(7)^. PSS statements have their response options ranging from 0 to 4 (0 = never to 4 = always) with a total score ranging from 0 to 56 points. Higher scores indicate greater perceived stress^(7)^. PSS internal consistency was assessed using Cronbach’s alpha (α) and presented a very good internal consistency index (α = 0.91).

The LMS, developed by Pirson et al.^(10)^, assesses the state of socio-cognitive mindfulness and has 21 items^(10)^. It was translated from American English and into Brazilian Portuguese^(8)^, and its use in the present study was authorized by the original author. Mindfulness can be understood as an active mindset characterized by a new perception of events that results in being (1) situated in the present, (2) sensitive to context and perspective, and (3) guided but not governed by rules and routines. In this regard, mindfulness manifests itself in cognitive flexibility that increases the degree to which an individual is seeking out new perspectives, engaging in creative activities, and the ability to engage with the present moment^(10)^. The LMS statements offer responses on a Likert-type scale ranging from 1 to 7 (1 = totally disagree to 7 = totally agree). The sum of item responses is the overall LMS score, which can range from 21 to 147 points^(10)^. The higher the value, the higher the level of mindfulness. The LMS showed good internal consistency (α = 0.80).

### Data Analysis and Treatment

The collected data were transferred to Microsoft Excel spreadsheets. After exclusion of participants’ email addresses by the main researcher, data were transferred to version 27.0 of the Statistical Package for the Social Sciences (SPSS) software for initial analysis and hypothesis testing. The R software was also used through the Rstudio interface and the ggstatsplot package (General Public License, v3.0) for further analysis. Descriptive analyzes of sociodemographic data, levels of mindfulness and quality of life were performed. In preliminary analysis, the frequency distributions of all variables were examined using the Shapiro-Wilk test.

The relationship of sociodemographic and health variables with the LMS and PSS results showed a non-normal distribution and were tested for the significance of group differences by non-parametric analysis of variance, Mann-Whitney and Kruskal-Wallis tests. In the analysis of the correlations between the perceived stress level and the total mindfulness score, there was a normal distribution. Pearson’s correlation test was applied and analyzed according to Cohen’s conventions for statistical power analysis: small (0.10 – 0.29), medium (0.30 – 0.49) and large (0.50 – 1.00)^(11)^. The significance level adopted in the study was 0.05.

### Ethical Aspects

The study was approved by the Research Ethics Committee with opinion number 4,250,872 of 2020, following the Resolution 466/2012 recommendations and with voluntary participation. In the initial stage of the research, volunteers had access to the ICF with instructions on the nature and objectives of the study. Only after expressing acceptance of participation in the research with the electronic ICF, the questionnaires were released to be answered.

## RESULTS

### Sociodemographic and Health Characterization

From the disclosure of the link to collection instruments, 1,024 individuals responded to the ICF made available in the first stage of collection. Of these, 13 (1.3%) chose to mark the item that they did not agree to participate in the survey, 47 (4.6%) responses were duplicated, 5 (0.5%) clicked on participate, but did not respond to the questionnaires. Four (0.4%) participants who left more than 20% of questions blank were also excluded. Thus, the final sample of this study consisted of 955 participants.

Of the 955 participants, most were from the state of Maranhão with 831 (87.0%); 622 (65.1%) were women; 465 (48.7%) were aged between 21 and 30 years old; 600 (62.8%) declared having non-white skin color; 699 (73.2%) had some religious belief; 509 (53.3%) had incomplete higher education; 392 (41.5%) had a monthly income of 1 – 3 minimum wages; 692 (72.9%) did not live with a partner; 725 (80.8%) had no children; and 584 (63.8%) were not working from home. Regarding the feeling of overload being greater at the time of the COVID-19 pandemic than before, the majority 409 (42.8%) stated that they completely agreed with the feeling of overload (Table 1).

**Table 1.** Participants’ sociodemographic variables (n = 955), Imperatriz, MA, Brazil, 2021.

Sociodemographic variables		n (%)
Sex (n = 952)	Female	622 (65.1)
	Male	333 (34.9)
Age group (years)	<21	192 (20.1)
	21 – 30	465 (48.7)
	31 – 40	168 (17.6)
	41 – 50	81 (8.5)
	51 – 60	37 (3.9)
	≥61	12 (1.3)
Skin color (n = 944)	White	344 (36.0)
	Non-white	600 (62.8)
Religious belief (n = 951)	Believer	699 (73.2)
	Non-believer	252 (26.4)
Education	Complete elementary school	4 (0.4)
	Incomplete high school	6 (0.6)
	Complete high school	101 (10.6)
	Incomplete higher education	509 (53.3)
	Complete higher education	110 (11.5)
	Specialization	87 (9.1)
	Masters’ degree	66 (6.9)
	Doctoral degree	72 (7.5)
Monthly income (minimum wages)	<1	151 (16.0)
	1 – 3	392 (41.5)
	3 – 6	201 (21.3)
	6 – 10	169 (17.9)
	11 – 15	31 (3.3)
State of residence	Maranhão	831 (87.0)
	Goiás	34 (3.6)
	Rio Grande do Norte	33 (3.5)
	Pará	10 (1.0)
	São Paulo	10 (1.0)
	Other states	37 (3.7)
Live with partner	Yes	257 (27.1)
	No	692 (72.9)
Children	0	725 (80.8)
	1	106 (11.8)
	2	66 (7.4)
Home office work	Yes	331 (36.2)
	No	584 (63.8)
I feel more overwhelmed today than before social isolation	(1) Totally agree	409 (42.8)
	(2) Partially agree	368 (38.5)
	(3) Partially disagree	113 (11.8)
	(4) Strongly disagree	65 (6.8)

Source: survey data.

With regard to health variables, the majority reported not having or living with people with chronic diseases (78.6% and 59.1%, respectively). A total of 390 (40.9%) of the sample stated that a family member had already been infected with COVID-19, and 504 (52.9%) respondents knew someone close to them who died as a result of COVID-19. Of the participants, 813 (85.1%) did not undergo psychotherapeutic follow-up; 324 (56.2%) did not drink frequently; 871 (91.5%) did not smoke; and 863 (90.4%) did not use psychotropic drugs during the period of the COVID-19 pandemic (Table 2).

**Table 2.** Distribution of participants according to health variables (n = 955), Imperatriz, MA, Brazil, 2021.

Health variables		N (%)
Chronic disease	Yes	203 (21.4)
	No	744 (78.6)
Live with someone with a chronic illness	Yes	389 (40.9)
	No	561 (59.1)
Who had COVID-19	Me	221 (23.2)
	Family	390 (40.9)
	Others	299 (31.3)
	Do not know	44 (4.6)
Someone close has died from COVID-19	Yes	504 (52.9)
	No	448 (47.1)
Psychotherapeutic follow-up	Yes	142 (14.9)
	No	813 (85.1)
Frequency of alcohol consumption	Once a month or less	324 (56.2)
	2 to 4 times a month	195 (33.8)
	2 to 3 times a week	51 (8.8)
	4 or more times a week	7 (1.2)
Smoke	Yes	81 (8.5)
	No	871 (91.5)
Use psychotropic	Yes	92 (9.6)
	No	863 (90.4)

Source: survey data.

### Comparison of Perceived Stress and Mindfulness with Sociodemographic and Health Variables

All participants responded to PSS. and the overall score was 32.2 (±9.8). A total of 952 individuals responded to LMS, with a total score of 94.4 (±14.5).

Table 3 shows the relationship between participants’ sociodemographic and health variables and the results of LMS and PSS regarding the significance of group differences by nonparametric analysis of variance, allowing a statistical analysis of significance of quantitative variables and nominal and ordinal qualitative variables.

**Table 3.** Relation of participants’ sociodemographic and health variables with the results of LMS and PSS, (N = 955), Imperatriz, MA, Brazil, 2021.

Sociodemographic and health variables		PSS (n = 955)		LMS (n = 952)	
		**Median** **(max – min)**	***p***	**Median** **(max – min)**	***p***
Sex (n = 952)	Female	34 (56 – 5)	<0.00*	93 (132 – 35)	<0.00*
	Male	30 (53 – 5)		98 (132 – 50)	
Age group (years) (n = 955)	<21	36 (55 – 6)	<0.00**	94 (123 – 50)	<0.00**
	21 – 30	35 (56 – 5)		92 (132 – 35)	
	31 – 40	30 (53 – 6)		95 (124 – 55)	
	41 – 50	28 (51 – 5)		101(130 – 67)	
	51 – 60	25 (43 – 8)		101 (132 – 50)	
	≥61	25 (39 – 9)		100 (129 – 78)	
Skin color (n = 944)	White	32 (56 – 5)	0.32*	96 (130 – 55)	0.02*
	Non-white	33 (56 – 5)		94 (132 – 35)	
Religious belief (n = 951)	Believer	32 (56 – 5)	<0.00*	94 (132 – 50)	0.04*
	Non-believer	36 (56 – 7)		97 (132 – 50)	
Education	Complete elementary school	34 (44 – 29)	<0.00**	87 (91 – 72)	<0.00**
	Incomplete high school	43 (48 – 16)		97 (103 – 71)	
	Complete high school	33 (53 – 5)		91 (123 – 50)	
	Incomplete higher education	35 (56 – 6)		93 (130 – 53)	
	Complete higher education	31 (53 – 11)		94 (126 – 63)	
	Specialization	30 (51 – 5)		96 (132 – 35)	
	Masters’ degree	30 (45 – 8)		99 (132 – 65)	
	Doctoral degree	28 (45 – 5)		103 (135–65)	
Monthly income (minimum wages)	<1	34 (56 – 6)	<0.00**	91 (129 – 55)	<0.00**
	1 – 3	34 (56 – 5)		95 (132 – 35)	
	3 – 6	33 (55 – 8)		95 (132 – 50)	
	6 – 10	30 (56 – 5)		98 (130 – 63)	
	11 – 15	28 (45 – 8)		98 (129 – 73)	
Live with partner	Yes	30 (54 – 5)	<0.00*	95 (132 – 56)	0.04*
	No	34 (56 – 5)		95 (132 – 35)	
Children	0	34 (56 – 5)	<0.00**	95 (132 – 50)	0.10**
	1	29 (50 – 5)		95 (121 – 35)	
	2	27 (50 – 11)		98 (126 – 64)	
Home office work	Yes	31 (55 – 5)	<0.00*	98 (130 – 35)	<0.00*
	No	34 (56 – 5)		92 (132 – 53)	
I feel more overwhelmed than before social isolation	(1) Totally agree	35 (56 – 6)	<0.00**	97 (132 – 35)	0.48**
	(2) Partially agree	31 (53 – 6)		94 (132 – 56)	
	(3) Partially disagree	30 (49 – 7)		94 (129 – 53)	
	(4) Strongly disagree	25 (53 – 5)		94 (130 – 50)	
Chronic disease	Yes	34 (56 – 9)	0.10*	97 (129 – 55)	<0.00*
	No	33 (56 – 5)		94 (132 – 35)	
Live with someone with a chronic illness	Yes	34 (56 – 6)	<0.00*	95 (132 – 50)	0.52*
	No	31 (56 – 5)		95 (130 – 35)	
Who had COVID-19	Me	35 (56 – 6)	<0.00**	94 (129 – 35)	0.38**
	Family	34 (55 – 5)		95 (132 – 55)	
	Others	31 (54 – 6)		95 (132 – 59)	
	Do not know	32 (52 – 14)		95 (128 – 67)	
Psychotherapeutic follow-up	Yes	35 (56 – 13)	<0.00*	99 (130 – 55)	0.01*
	No	32 (56 – 5)		94 (132 – 35)	
Use psychotropic	Yes	34 (56 – 13)	0.03*	95 (129 – 55)	0.93*
	No	33 (56 – 5)		95 (132 – 35)	
Smoke	Yes	34 (54 – 11)	0.01*	94 (124 – 63)	0.49*
	No	32 (56 – 5)		95 (132 – 35)	

PSS: Perceived Stress Scale; LMS: Langer Mindfulness Scale. *Mann-Whitney U Test. **Kruskal–Wallis test, p-value < 0.05. Source: survey data.

According to Table 3, the comparison of sex, age group, religious belief, education level, monthly income, living with a partner, home office work, feeling overwhelmed, living with someone with a chronic illness, who had COVID-19 and psychotherapeutic follow-up with the assessment of perceived stress (PSS) variables. Also, statistically significant (p < 0.00) were the comparison of sex, age group, religious belief, monthly income, home office work and chronic illness with the assessment of mindfulness (LMS).

### Correlation Between Perceived Stress and Mindfulness

**Figure 1.**
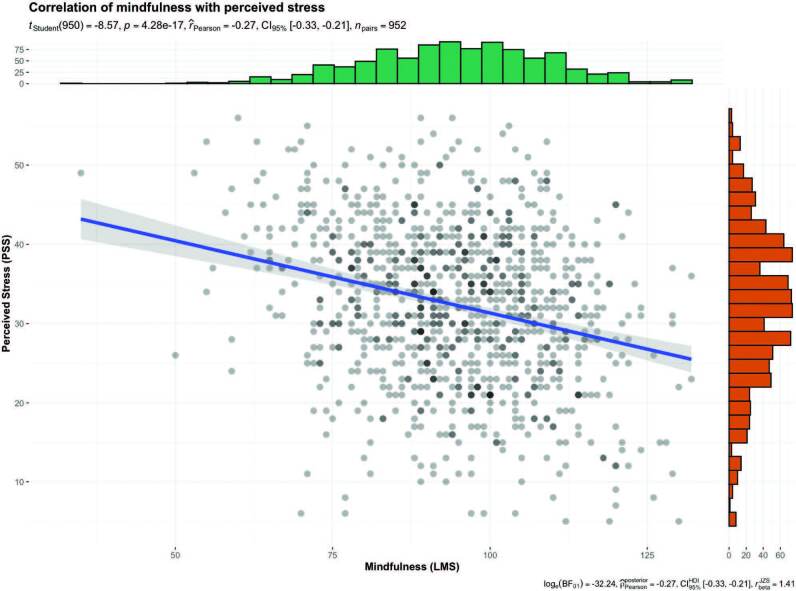
Correlation between mindfulness (LMS) and perceived stress (PSS). PSS: Perceived Stress Scale; LMS: Langer Mindfulness Scale. The distribution of each variable is shown on the graph’s diagonals. In the central part of the figure, a bivariate scatter plot is displayed with a fitted line indicating the direction of the relationship between variables. Of the sample of 955, three failed to respond to the LSM in full. Method: Pearson Source: Survey data using statistical package ggstatsplot package.

Pearson’s correlation showed that in 952 participants, the mindfulness variable (LMS) showed a negative correlation with perceived stress (PSS): t(950) = –8.57, p = 4.28e–17), i.e., the greater the mindfulness, the lower the perceived stress. The effect size (r = –0.27, 95%CI[–0.33, –0.21]) was small as per Cohen’s conventions. The Bayes Factor (loge(BF01) = –32.24, r = –0.2795%CI[–0.33, –0.21]) for the same analysis revealed strong evidence that the results indicated that perceived stress had no statistically significant association with mindfulness in this study’s sample (Figure 1).

## DISCUSSION

Overall, the results of this survey showed that most participants had general perceived stress scores ranging from moderate (59.1%) to high (32%) and mindfulness with high levels (44.8%). The factors that most influenced these results were sex, age group, religious beliefs, monthly family income, living with a partner, type of work and psychotherapeutic follow-up.

In this research, women had higher levels of perceived stress when compared to men. Although these findings demonstrate that the pandemic has not altered these differences between men and women, studies have found a strong relationship between the female and the experience of loneliness during the COVID-19 pandemic^(12)^ and a greater propensity for women to be afraid of COVID-19^(13)^. In these studies, loneliness and fear were factors that influenced stress levels in women during the COVID-19 pandemic, due to the need for physical isolation and the risk of illness^(12,13)^.

Mindfulness levels were higher in older males, but the literature is still scarce in terms of studies describing the relationship between sociodemographic characteristics and sociocognitive mindfulness, with a greater range of studies related to meditative mindfulness^(14)^. Resilience may be one of the factors that explain higher levels of mindfulness in older people, especially the ability to produce different perspectives and involvement with current events^(8,15)^.

Participants who reported lower income and completed primary and secondary education had higher levels of perceived stress. During the COVID-19 pandemic, an adequate family income and a good educational background were protective factors for mental health by allowing better processing of information and the economic instability caused during the crisis^(16)^. On the other hand, financial insecurity, the inability to deal with the excess of false information, the extensive media coverage of COVID-19, death and misfortune have contributed to increasing the population’s anguish^(16)^.

According to the results of this study, individuals without religious belief had higher levels of perceived stress and higher levels of mindfulness. Interpreting these findings according to the socio-cognitive attention theory^(10)^, it is likely that the context of the COVID-19 pandemic has encouraged novelty and flexibility production in non-religious people, because these characteristics are related to the way a person moves in their environment^(10)^. The tendency to produce novelties leads to an ability to act in the environment by actively creating new categories instead of depending on the usual way of dealing with everyday situations. Flexibility is the ability to consciously observe one’s experiences from different perspectives, causing necessary changes in one’s behavior^(17)^.

In this study, single people without children had higher levels of stress, which contrasts with the results of a survey of 53,524 internet users conducted in 26 countries^(18)^ showing that individuals with children reported higher levels of stress during the pandemic of COVID-19 compared to people living alone or with an adult.

However, the higher levels of stress in single people without children may be in line with the literature by showing that the absence of family relationships deprives the individual of the opportunity for social interaction, especially in times of physical isolation, which in turn has the potential to impact on biopsychosocial well-being^(19)^.

It is known that having children can be a stressful factor due to the overload of domestic services, the fear that children may contract COVID-19 and even higher economic costs. These issues may increase perceived stress levels in people with more children at home^(18)^. However, our findings showed that individuals with two children at home had lower levels of stress during the COVID-19 pandemic. It is possible that the well-being of children with siblings was less affected when compared to only children, because having someone to play with during the period of physical distancing contributed to children’s well-being^(20)^.

Participants who were working from home achieved higher degrees of mindfulness. Working from home in times like the COVID-19 pandemic has the potential to promote benefits such as a lower risk of infection and a greater sense of security. These benefits may be related to an increased state of mindfulness^(21)^.

However, individuals who strongly agreed with feeling more overwhelmed at the time of the survey than before the COVID-19 pandemic, had higher levels of stress. Regarding overload in this period, the main findings are related to technological means. People who reported greater development of asynchronous activities using digital tools had a greater perception of overload, while the development of synchronous activities was not associated with overload^(22)^.

From the analysis of health variables, it was noticed that those who had a chronic disease obtained a higher level of mindfulness, despite this population being included in the risk group during the COVID-19 pandemic^(23)^. The experience of living with a chronic disease may have contributed to the development of greater attention to current events and greater self-care skills in this group, which may favor the state of mindfulness^(24)^.

The results that indicated higher levels of stress in individuals who claimed to have contracted COVID-19 and who lived with people with a chronic disease may be related to insecurity about health conditions and the large flow of insufficient news to provide security for the disease control. These factors were considered potential triggers for both stress and post-traumatic stress disorder^(25)^. Although stress is a condition present in those who live with or are caregivers of people with chronic diseases^(23)^, the pandemic context may have increased this condition. In line with our findings, a survey of 1,210 people in China showed a moderate to severe psychological impact on more than half of respondents, with 75.2% concerned about the possibility of family members having COVID-19^(26)^.

Stressors related to the COVID-19 crisis will impact the general population’s mental health for an indefinite period, generating an expectation that at least 10.0% of the population will develop symptoms of post-traumatic stress^(27)^. In this context, psychotherapeutic monitoring is an important tool to help individuals deal with the mental health and behavioral consequences of the pandemic^(27)^. In our sample, the majority (85.1%) of participants answered that they were not undergoing psychotherapeutic follow-up at the time of research, as they had lower levels of mindfulness. This data shows the need to popularize and disseminate the importance of mental health care.

The correlations between general mindfulness assessment scores (LMS) and perceived stress (PSS) indicated that the greater the mindfulness, the lower the levels of perceived stress, which is similar to the findings of other studies in which mindfulness is positively associated with to quality of life and negatively to various psychological symptoms and perceived stress^(28)^. However, in this study, sociocognitive mindfulness did not show a statistical difference in the sample in the context of the COVID-19 pandemic. This was probably due to the fact that LMS is structured around novelty perception constructs^(10)^ and the pandemic was a period of rapid social and daily transformations that were necessary to promote an adaptation that represented a real need to preserve life.

Identifying possible risk factors for mental health in the general population during the COVID-10 pandemic through an assessment of participants’ levels of socio-cognitive mindfulness and perceived stress was important to better understand the crisis process faced by the population. Sudden changes caused by COVID-19 brought feelings of lack of control and unpredictability that required adaptability through attention to what was happening and sensitivity to the pandemic context^(9,10)^.

As for the limitations of this research, firstly, online data collection may be at risk of bias, as not all people have equal chances of receiving an invitation to participate. Secondly, we recognized the sample’s non-probabilistic characteristic, which was mostly concentrated in the northeast region, even though the questionnaire was disseminated in groups from all regions of the country. However, this limitation may have occurred due to the fact that one of the tools used that enabled a broader dissemination of the questionnaires was through the Integrated System of Management of Academic Activities of UFMA, allowing the questionnaires to have a greater reach, mainly to UFMA students and employees in its various poles. Although we do not have data that indicate the frequency distribution of this type of study in the regions of the national territory, hypothetically there may be a relationship between availability and the number of invitations to participate voluntarily.

Moreover, the current research assessed specific aspects of a specific moment amidst the COVID-19 pandemic, with no comparison of the psychic state in moments prior to the pandemic. Furthermore, such issues suggest the need for further studies in order to assess the extent of related results in other populations.

## CONCLUSIONS

This research made it possible to verify the risk factors for mental health, the levels of mindfulness and perceived stress of individuals in the context of the COVID-19 pandemic. In particular, female participants and individuals with low socioeconomic conditions were the most affected by the COVID-19 pandemic, showing higher levels of stress. On the other hand, psychological support, the presence of children at home, family relationships and a religious belief can be protective factors.

Although the relationship between mindfulness and lower levels of stress is consolidated in the literature, sociocognitive mindfulness measurement showed that this perspective strongly influenced by social constructs and novelties was not a protective factor for perceived stress in the context of the COVID-19 pandemic.
